# Impact of parental migration on oral health outcomes of left-behind school-aged children in Luchuan, southern China

**DOI:** 10.1186/s12903-018-0683-3

**Published:** 2018-12-11

**Authors:** Rongmin Qiu, Yihong Li, Manisha Malla, Junyu Yao, Dan Mo, Neha Dhakal, Hua Huang

**Affiliations:** 1 Department of Pediatric Dentistry, College &Hospital of Stomatology, Guangxi Key Laboratory of the Rehabilitation and Reconstruction of Oral and Maxillofacial Research, Guangxi Key Laboratory of Treatment and Research for Oral and Maxillofacial Surgery Disease, Guangxi Medical University, 10 Shuangyong Road, 530021, Nanning, Guangxi Province China; 2 Department of Basic Science Craniofacial Biology, College of Dentistry, New York University, New York, 10010 USA

**Keywords:** Parental migration, Left-behind children, Dental caries, Oral health related behaviors, School-aged children

## Abstract

**Background:**

With rapid urbanization in China, an increasing number of rural adults have migrated to cities to seek job opportunities, leaving their school-aged children behind. These left-behind children (LBC) without one or both parents usually receive less attention from their caregivers. Whether the parental migration affects the children’s oral health is not well understood. This study aimed to explore the differences in dental caries status and oral health-related behaviors between children with different parental migration experiences in a rural area of Southern China.

**Methods:**

A cross-sectional study was conducted in Luchuan County of Guangxi Province in 2015. A total of 1085 school children aged 8–12 participated in this study. Participants’ demographic characteristics, parental migration information, and eating and oral hygiene habits were collected using a self-administered questionnaire. Dental caries of permanent teeth was examined using the decayed, missing, and filled tooth (DMFT) index recommended by the World Health Organization. Dental caries experience and oral health-related behaviors were compared between LBC and non-LBC, as well as children with different experiences of parental migrations. The impact of various parental migration attributes on LBC oral health outcomes was examined by univariate and multivariate analyses.

**Results:**

Among the school-aged children examined, 60.9% of them were LBC. Only 29.7% of the children brushed their teeth regularly; 86.5% of them did not know what fluoride toothpaste was. Caries prevalence was 51.4% for LBC and 40.8% for non-LBC (*p* < 0.001). The LBC experienced a greater DMFT mean (1.20 ± 1.59) compared to the non-LBC (0.85 ± 1.30) (*p* < 0.001). Oral health-related behaviors were not significantly different between LBC and non-LBC. Dental caries experience and oral health-related behaviors were not related to the type or duration of parental migration. Multiple regression analyses showed that parental migration was one of significant predictors of children’s caries outcome; LBC had a higher risk to caries than non-LBC (*95*% CI =1.26, 2.09).

**Conclusions:**

These findings indicate that parental migration could be a significant risk factor for caries development among 8- to 12-year-old school children in rural China.

**Electronic supplementary material:**

The online version of this article (10.1186/s12903-018-0683-3) contains supplementary material, which is available to authorized users.

## Background

Since 1979, China has been undergoing rapid economic development and urbanization. An increasing number of rural adults have migrated to cities to seek job opportunities and better income to support their families. Meanwhile, children of those families usually do not follow their parents to the cities due to the restrictions of the Chinese census registration system and personal financial constraints [1]. As a result, many children have been left behind in the countryside. These children are usually called left-behind children (LBC). It was estimated that more than 61 million children nationwide were left behind in 2010, accounting for 37.7% of the total rural children population and 21.9% of the total population of children in China. The number of LBC children, most of them preschool and school-aged children, has increased another 2.42 million from 2005 to 2010; one-third of LBC were primarily taken care of by their grandparents or relatives [2, 3]. A significant portion of the LBC live in the central and southern regions of China [2], including Guangxi rural areas from where people migrant to Guangdong Province, the economic development zone. Since the implementation of economic reformation and open-door policies, thousands of adults have migrated from the countryside to metropolitan areas seeking a better life. It was reported that nearly 1.4 million LBC lived in the rural area of Guangxi in 2014, and 65.9% of them were school-aged children [4].

Parents play a key role in children’s physical and psychological health development. However, parental migration usually leads to inadequate parental care of the children, resulting in physical, psychological and even social health consequences among LBC [5]. As most primary caregivers of LBC were grandparents who did not complete middle school education, they are unaware of the importance of immunization to a child’s health; hence, the grandparents often failed to follow the immunization schedules, leading to a high prevalence of delayed vaccination among LBC aged 12–72 months [6]. LBC are often fed food with a low nutritional value or high in carbohydrates [7], predisposing those children to a relatively higher risk of wasting, obesity, and being overweight [8]. Additionally, without sufficient parent-child communication, LBC were likely to suffer from a broad range of psychological health problems, such as depression, anxiety, and loneliness [9–11]. Evidence even suggests that LBC experience poorer health-related quality of life than non-LBC due to psychosocial dysfunction [12]. In addition to physical problems and psychological maladjustments, a previous study showed that parental migration could cause a series of unhealthy behavioral problems among LBC and adolescents, such as Internet addiction, tobacco smoking, and drinking excessive amounts of sweetened beverages [13]. In addition, LBC who perceived emotional neglect from their parents had an increased risk of suicide attempts [14]. Thus, LBC are more vulnerable and may encounter more difficulties in their lives if their parents are absent.

Dental caries, known as a common chronic childhood disease around the world, are the primary pathological cause of tooth loss in children [15]. The most current Chinese National Oral Health Survey data show that caries prevalence in permanent dentition was 34.5% in 2015 among children of 12 years of age in China, a 7.8% increase compared to that in 2005 [16]. Among 12-year-old children who lived in the Guangxi rural area, 60.5% of them had dental caries [17]. The prevalence was twice as high as the national average. It was hypothesized that a lack of oral health knowledge, less access to effective caries prevention programs, and inadequate dental care services in the Guangxi general population contribute to the high caries prevalence. Caries development is closely associated with poor oral health habits, such as frequent sugar or soft drink intake, not brushing teeth regularly, no exposure to fluoride, and inadequate oral health care [18].

Early establishment of good oral health habits at a young age is crucial to reduce caries risk and prevent disease. Listl, et al. demonstrated that children who grow up with their non-biological parents consumed more sugar, juice, cake, cookies, and chocolate then children grow up with their nature parents. They experienced more caries, but were less likely to access dental services [19]. Thus, adopting consistent healthy behavioral habits in childhood occurs at home and caregivers’ good oral health practice can be the primary model for children’s behavior establishment. However, most LBC caregivers are grandparents older than 60 years old [3], and they have limited knowledge of oral health or how to teach the children better oral health behaviors. The present study aimed to investigate the impact of parental migration on dental caries and oral health-related behaviors among LBC aged 8–12 in Luchuan County, Guangxi Province, China. A caries status examination and a questionnaire survey on oral health-related behaviors were conducted. The hypothesis of the study was that LBC experienced poor oral health outcomes due to parental migration compared to their non-LBC counterparts who lived in the same rural areas.

## Method

### Study participants

A cross-sectional study was conducted from July to December of 2015 in four randomly selected secondary schools in the rural area of Luchuan County, Guangxi Province in Southern China. Luchuan has a population of 109.5 million and a rural population of 75.24 million (68.7%). The estimated per capita annual net income was 9153 RMB in 2014 for the rural population, which was slightly higher than the provincial average of 7803 RMB (2014) [20]. The county was divided into 14 administrative districts. There were approximately 0.86 million children enrolled in primary schools in 2015. The sample size for this study was determined based on a previously reported caries prevalence of 60.5% among 12-year-old children in the Guangxi rural area [17]. Therefore, a minimum required sample size was 1044 school-aged children.

To obtain a representative sample size, a two-stage sampling technique was employed. In the first stage, four of the 14 administrative districts were randomly selected in Luchuan County. In the second stage, a cluster sampling method was performed. One primary school in each selected administrative district was selected. All of the children aged 8–12 in the four-selected school were invited to participate in the study. The response rate was 99.1%. A written-informed consent was obtained from each child’s caregiver in advance. Children who were orphans, of single-parent families, could not answer the questionnaire. Ten children who did not cooperate with an oral examination were excluded. Finally, a total of 1085 school children were enrolled in the study.The study protocol was approved by the Ethics Committee of College of Stomatology, Guangxi Medical University.

### Data collection

#### Information on parental migration experience

In the study, the LBC were defined as children who were under 18 years of age, had been left behind at their original residence while one or both of the parents migrated to other places for work and were not cared for by their biological parents [3]. Two questions related to parental immigration were evaluated: 1) children were asked about which of their parents migrates to other places for work (father-only, mother-only, both parents, and none of the parents), and 2) for the children with parental migration, they were asked how often their parents came back home (≤3 months, 4–6 months, 7–12 months, 1–2 years, and > 2 years).

#### Questionnaire survey

A questionnaire survey was conducted with the children first, based on the fourth Chinese National Oral Health Survey Methods [16]. Information collected included the following: 1) participates’ demographic background, including children’s gender and age; 2) parental migration experience; 3) children’s eating habits, including intake frequency of sugary snacks, beverages, and drinks with added sugar; and 4) children’s oral health behaviors, including tooth brushing habits, use of fluoride toothpaste, and oral health education history. The questionnaire was pilot tested and validated among 120 8- to 12-year-old children prior to the study. In the study, the questionnaires were distributed to all children in their classrooms and completed under the supervision of the investigators.

#### Caries examination

Clinical examinations were performed in the classroom under natural light with the children lying on a desk and the examiner seated on a chair behind the subject. The examiner was trained and calibrated for dental caries diagnosis based on the World Health Organization (WHO) Health Survey Methods for field studies [21]. Caries status was recorded using the decayed, missing, and filled tooth (DMFT) index for permanent teeth. An intra-examiner calibration was performed weekly. The intra-examiner kappa values for DMFT scores were > 0.85.

### Data analysis

The main outcome of the study was children’s caries status (caries prevalence and DMFT score), and the primary independent variables included parental migration (LBC verses non-LBC) and type and duration of parental migration. The secondary assessed variables were children’s oral health-related behaviors. The main testing hypothesis was that parental migration experience, including parental migration and the type and duration of parental migration would influence children’s oral health behavior, thus resulting in an increased risk for dental caries. In the analysis, caries experience was dichotomized as absent or present and the disease severity was reported based on DMFT score and treated as a continuous variable. Analysis of variance (ANOVA), *Chi-square* test, and non-parametric test were used to examine and compare the distribution of the demographic variables, and all other categorical and continuous variables were examined between LBC and non-LBC, various types of parental migration, and durations of parental migration experience (dichotomized as ≤6 months and > 6 months). Multivariate logistic regression analyses were used to examine potential relationship between the parental migration experience and caries prevalence as well as the oral health-related behaviors among the left-behind children, controlling demographic and other potential confounding variables. All data analyses were processed via SPSS statistical software v.24.0 (IBM, Armonk, NY, US). All tests were two-sided, and *p* < 0.05 was considered significant.

## Results

In total, 1085 school children aged 8–12 participated (49.4% boys and 50.6% girls) in this study, and their average age was 10.06 ± 1.35 years. Among these children, 60.9% (*N* = 661) of them were LBC, and 39.1% (*N* = 424) of them were non-LBC. Among the LBC, one-third of them lived without fathers and more than half of them lived without both parents, and 44.2% of their migrant parents left for more than 6 months (Table 1). Almost one-third of the children consumed sugary snacks or sweet beverages frequently (equal to or more than once per day). Only 29.7% of the children brushed their teeth twice per day; 86.5% of them did not know what fluoride toothpaste was. None of the children had received oral health education (Table 1).

**Table 1:** Characteristics of the study population (*N* = 1085)

Variables	Frequency (N)	Percentage (%)
Gender		
Boy	536	49.4
Girl	549	50.6
Age (years old)		
8–9	393	36.2
10	258	23.8
11–12	434	40.0
Parental migration		
LBC	661	60.9
Non-LBC	424	39.1
Type of parental migration (N = 661)		
Father-only migration	213	33.2
Mother-only migration	89	13.5
Both parent migration	359	54.3
Duration of parental migration (N = 661)		
≤ 6 months	369	55.8
> 6 months	292	44.2
Frequency of sugary snack intake		
< once/day	750	69.1
≥ once/day	335	30.9
Frequency of sugary beverages intake		
< once/day	789	72.7
≥ once/day	296	27.3
Frequency of drink^a^ with added sugar intake		
< once/day	845	77.9
≥ once/day	240	22.1
Tooth brushing frequency		
Not every day	105	9.7
Once/day	658	60.6
≥ twice/day	322	29.7
Fluoride toothpaste use		
Yes	74	6.8
No	73	6.7
Unknown	938	86.5
Having received oral health education or not		
Yes	0	0
No	1085	100

^a^Milk, tea, coffee, soybean milk

Overall, caries prevalence in permanent dentition of the 1085 children was 47.3% with a mean DMFT score of 1.06 (SD ± 1.50). The study also found that female children and older children experienced more dental caries (*p* < 0.001) compared to male and younger children (Table 2). The LBC group showed a significantly higher caries prevalence compared to the non-LBC group (51.4% vs. 40.8%; *p* < 0.001) (Table 2). The LBC group also experienced higher mean DMFT scores than the non-LBC group (1.20 ± 1.59 vs. 0.85 ± 1.30; *p* < 0.001). In addition, children without a father or both parents present experienced more caries compared to the non-LBC group (Table 2). Interestingly, there were no significant differences between various parental migration experiences and children’s gender, age, dietary habits, or oral health practice (Table 3).

**Table 2:** Dental caries experience of the study population (N = 1085)

Variables	Dental caries experience				
	Absent	Present		DMFT Score	
	N (%)	N (%)	*p* value*	Mean ± SD	*p* value^†^
Total	572 (52.7)	513 (47.3)		1.06 ± 1.50	
Gender					
Boy	313 (58.4)	223 (41.6)	< 0.001	0.86 ± 1.28	< 0.001
Girl	259 (47.2)	290 (52.8)		1.26 ± 1.65	
Age (years)					
8–9	250 (63.6)	143 (36.4)	< 0.001	0.69 ± 1.16	< 0.001
10	121 (46.9)	137 (53.1)		1.12 ± 1.46	
11–12	201 (46.3)	283 (53.7)		1.36 ± 1.70	
Parental migration					
Non-LBC	251 (59.2)	173 (40.8)		0.85 ± 1.30	
LBC	321 (48.6)	340 (51.4)	< 0.001	1.20 ± 1.59	< 0.001
Type of parental migration					
Non-LBC	251 (59.2)	173 (40.8)	0.004	0.85 ± 1.30	0.002
Father-only migration	100 (46.9)	113 (53.1)		1.23 ± 1.48	
Mother-only migration	49 (55.1)	40 (44.9)		1.20 ± 1.91	
Both parent migration	172 (47.9)	187 (52.1)		1.17 ± 1.58	
Duration of parental migration					
Non-LBC	251 (59.2)	173 (40.8)	0.001	0.85 ± 1.30	< 0.001
≤ 6 months	169 (45.8)	200 (54.2)		1.24 ± 1.56	
> 6 months	152 (52.1)	140 (47.9)		1.14 ± 1.64	
Frequency of sugary snack intake					
< once/day	399 (53.2)	351 (46.8)	0.635	1.17 ± 1.50	0.917
≥ once/day	173 (51.6)	162 (48.4)		1.04 ± 1.48	
Frequency of sugary beverages intake					
< once/day	405 (51.3)	384 (48.7)	0.135	1.10 ± 1.50	0.100
≥ once/day	167 (56.4)	129 (43.6)		0.96 ± 1.48	
Frequency of drink with added sugar intake					
< once/day	438 (51.8)	407 (48.2)	0.273	1.10 ± 1.53	0.170
≥ once/day	134 (55.8)	106 (44.2)		0.93 ± 1.39	
Tooth brushing frequency					
Not every day	62 (59.0)	43 (41.0)	0.386	0.89 ± 1.34	0.900
Once/day	341 (51.8)	317 (48.2)		1.08 ± 1.53	
≥ twice/day	169 (52.5)	153 (47.5)		1.09 ± 1.48	

*Chi-square test

^†^Non-parametric Kruskal-Wallis test

**Table 3:** Comparisons of oral health-related variables between various experiences of parental migration (N = 1085)

Variables	Parental migration		Types of migration			Duration of migration	
	Non-LBC N (%)	LBC N (%)	Father-only N (%)	Mother-only N (%)	Both parent N (%)	≤ 6 months N (%)	< 6 months N (%)
	424	661	213	89	359	369	292
Gender							
Boy	218 (51.4)	318 (48.1)	91 (42.7)	46 (51.7)	181 (50.4)	169 (45.8)	149 (51.0)
Girl	206 (48.6)	343 (51.9)	122 (57.3)	43 (48.3)	178 (49.6)	200 (54.2)	143 (49.0)
Age (years old)							
8–9	137 (32.3)	256 (38.7)	80 (30.8)	36 (40.4)	140 (39.0)	136 (36.9)	120 (41.1)
10	101 (23.8)	157 (23.8)	101 (38.7)	22 (24.7)	82 (22.8)	84 (22.8)	73 (25.0)
11–12	186 (43.9)	248 (37.5)	80 (30.8)	31 (34.8)	137 (38.2)	149 (40.4)	99 (33.9)
Frequency sugary snack intake							
< once/day	288 (67.9)	462 (69.9)	154 (72.3)	62 (69.7)	246 (68.5)	258 (69.9)	204 (69.9)
≥ once/day	136 (32.1)	199 (30.1)	59 (27.7)	27 (30.3)	113 (31.5)	111 (30.1)	88 (30.1)
Frequency of sugary beverages intake							
< once/day	299 (70.5)	490 (74.1)	159 (56.0)	65 (54.6)	266 (74.1)	280 (75.9)	210 (71.9)
≥ once/day	125 (29.5)	171 (25.9)	125 (44.0)	54 (45.4)	93 (25.9)	89 (24.1)	82 (28.1)
Frequency of drink with added sugar intake							
< once/day	332 (78.3)	513 (77.6)	152 (71.4)	73 (82.0)	288 (80.2)	283 (76.7)	230 (78.8)
≥ once/day	92 (21.7)	148 (22.4)	61 (28.6)	16 (18.0)	71 (19.8)	86 (23.3)	62 (21.2)
Tooth brushing frequency							
Not every day	33 (7.8)	72 (10.9)	23 (10.8)	10 (11.2)	39 (10.9)	41 (11.0)	31(10.6)
Once/day	259 (61.1)	399 (60.4)	115 (54.0)	59 (66.3)	225 (62.7)	216 (58.5)	183 (62.7)
≥ twice/day	132 (31.1)	190 (28.7)	75 (35.2)	20 (22.5)	95 (26.5)	112 (30.4)	78 (26.7)

All the groups with different experiences of parental migration were compared between LBC and non-LBC. All p-values were > 0.05. The comparisons within different experiences of parental migration were not statistically significant (All *p*-values > 0.05). The exact *p*-values for all the comparisons were listed in the Additional file 1: Table S1

After controlling for the demographic and other potential confounding factors, parental migration was significantly correlated to higher caries prevalence (*95*% CI =1.26, 2.09). No multicollinearity was detected among the independent variables in the regression models. In addition to the parental migration experience, the final multiple regression analysis model demonstrated that gender and age were also significant contributing factors to the increased risk for dental caries (*p* < 0.001). The final Hosmer-Lemeshow test with a *p-*value of 0.80 indicated that the final reduced model was a good fit in predicting caries outcome in the LBC population (Table 4).

**Table 4:** Summary of logistic regression analysis for children’s caries status in relation to parent’s migration status, children’s sugar consumption and oral health habit (N = 1085)

Variables	Caries status	ORs for caries status (95% CI)	
	Present (%)	Full model ^a^	Reduced model ^b^
Total	513 (47.3)		
Gender			
Boy	223 (41.6)	1.00 (referent)	1.00 (referent)
Girl	290 (52.8)	1.56 (1.22–2.00)	1.59 (1.24, 2.03)
Age (years old)			
8–9	143 (36.4)	1.00 (referent)	1.00 (referent)
10	137 (53.1)	2.08 (1.50, 2.89)	2.08 (1.51, 2.88)
11–12	283 (53.7)	2.09 (1.57, 2.73)	2.15 (1.62, 2.86)
Parental migration			
Non-LBC	340 (51.4)	1.00 (referent)	1.00 (referent)
LBC	173 (40.8)	1.52 (1.09, 2.11)	1.62 (1.26, 2.09)
Type of parental migration			
Non-LBC	173 (40.8)		
Father-only migration	113 (53.1)	1.00 (referent)	
Mother-only migration	40 (44.9)	0.97 (0.68,1.38)	
Both parent migration	187 (52.1)	0.72 (0.45, 1.17)	
Duration of parental migration			
Non-LBC	173 (40.8)		
≤ 6 months	200 (54.2)	1.00 (referent)	
> 6 months	140 (47.9)	1.26 (0.91, 1.74)	
Frequency sugary snack intake			
< once/day	351 (46.8)	1.00 (referent)	
≥ once/day	162 (48.4)	0.80 (0.59, 1.08)	
Frequency of sugary beverages intake			
< once/day	384 (48.7)	1.00 (referent)	
≥ once/day	129 (43.6)	1.13 (0.82, 1.56)	
Frequency of sugar added drink intake			
< once/day	407 (48.2)	1.00 (referent)	
≥ once/day	106 (44.2)	1.17 (0.84, 1.62)	
Tooth brushing frequency			
Not everyday	43 (41.0)	1.00 (referent)	
Once/day	317 (48.2)	1.18 (0.74, 1.88)	
≥ twice/day	153 (47.5)	1.18 (0.76, 1.82)	

^a^Full model summary: -2Log likelihood = 1435.627; Cox and Snell R^2^ = 0.058; Nagelkerke R^2^ = 0.078; Chi-square for the full model = 65.292; df = 12, *p* < 0.001; Chi-square for Hosmer and Lemeshow test = 7.585; df = 8; *p* = 0.475; the overall percentage correct = 59.5

^b^Reduced model summary: -2Log likelihood = 1442.658; Cox and Snell R^2^ = 0.052; Nagelkerke R^2^ = 0.070; Chi-square for the reduced model = 58.261; df = 4, *p* < 0.001; Chi-square for Hosmer and Lemeshow test = 3.818; df = 7; *p* = 0.800; the overall percentage correct = 59.4

## Discussion

This cross-sectional study showed that the prevalence of LBC was 60.9%, similar to the data among the school children population of Guangxi in 2014 (65.9%) [4], but higher than that in the previous studies [12, 13]. One explanation could relate to the use of different LBC definitions. Some studies have suggested that the definition of LBC includes parental migration of at least 6 months [12, 13]. The present study used the definition proposed by the China Women’s Federation without a time limitation for the parental migration [3]. Another reason might be the age differences between the present studies compared to previous studies in which older children were examined. The proportion of LBC among adolescent children is usually lower than that among primary school children [3]. Boys or older children usually followed their parents to work in the cities; girls or younger children were usually left behind at home [3]. In our study, there were no differences between genders and age groups between the LBC and non-LBC groups. More than half of LBC lived without both parents and need more attention paid to their health condition. The result was similar to the previous report [3].

The overall caries prevalence in permanent teeth was 47.3% in the study population of a rural area of Luchuan. The prevalence was lower than that in the survey in Guangxi [17]. In the previous survey, children aged 12, as the standard age-group by WHO, were examined representing the caries status of permanent dentition. In the present study, children aged 8–12 were included and the caries prevalence was significantly correlated with age increase. Girls were found to have higher caries prevalence and DMFT scores, a finding consistent with the previous study [17], and the girls most likely preferred a higher sweet consumption compared with boys.

Several studies have demonstrated that children with parental migration would be more prone to have physical and psychological problems [5–11]. In this study, we observed that left-behind children were at higher risk of dental caries in the rural area of Luchuan. The overall parental migration experience, rather than the type or duration of parental migration, was significantly associated with the higher caries prevalence and higher DMFT scores among the LBC. The findings suggest that parental migration could be an important contributing factor for the increased susceptibility for caries among LBC by means of direct (oral health neglect) or indirect (unhealthy oral health behaviors) actions. Parental migration has been considered a risk factor for unhealthy behaviors among school-aged LBC, such as cigarette smoking, excessive drinking of sweetened beverages or alcohol, and macronutrient intake [13, 22, 23]. The plausible pathways through which parental migration affects children’s health behaviors can be complex and multi-factorial. First, the outcomes may be related to oral health perception of the grandparents who are the main caregivers of the children left behind. A study by Zhang, et al. reported that the health knowledge and health behaviors of caregivers could influence the children’s health habits [22]. Second, the outcomes might be associated with children’s satisfaction by substances from their parents [24]. Parents who are absent from their children usually feel guilty and they may compensate the children with more pleasant substances, often containing added sugar. Third, increasing family income may be a contributing factor. National data have shown that income levels are higher for migrants than their rural counterparts [25]. In China, rising affluence has also been shown to increase unhealthy behaviors and contribute to obesity and being overweight, as children with higher family incomes are more likely to have opportunities for unhealthy choices [26, 27].

Luchuan County is known as a labor export county. The per capita annual net income in Luchuan was higher than the provincial average in 2014. Previous studies have reported that children who are raised by one parent, grandparents or other relatives are more likely to face health and social-behavioral developmental challenges [6, 22, 28, 29]. One of the study assumptions was that commercial sugary snacks and beverages are accessible and affordable to the general community, and therefore dietary factors and poor oral hygiene might influence the caries outcome, particularly among LBC. The results show that the LBC experienced more dental caries. However, whether one parent or two parents left home, the children still suffered from similar dental caries experiences and had similar oral health behaviors. Although no significant relationship was found between parental migration experience and oral health related behaviors, we found that children in this rural community have not received adequate oral health instruction. One in three children had sugary snacks or beverages regularly. Majority of the children have never used fluoride toothpaste. The development of caries is a complicated process, depending on many factors, such as tooth development defects, cariogenic microbial colonization, dynamics of the oral environment, dietary habits, oral health-related behaviors, and medical care [18]. Currently, the literature on the characteristics of parental migration and the impacts on children’s health are lacking. Whether and how the parental migration practice impacts children’s dental caries is still unclear; therefore, further studies are needed to elucidate the possible biological connections.

Although the study did not observe a significant relationship between parental migration experience and oral heath behaviors, a few points are worth noting. First, the study found that only 29.7% of children aged 8–12 brushed their teeth twice a day. The results are similar to the recently published national survey data, which showed that 24.1% of 5-year-old and 31.9% of 12-year-old children in the similar region brushed their teeth twice a day [16]. Second, the study found that 86.5% of children in Luchuan County did not know what fluoride toothpaste was and none of these children living in the rural area had received oral health education. Additionally, sugar consumption is a well-known risk factor for caries development [30, 31]. Children grow up with single biological parent or both non-biological parents showed different sugar consumption patterns compared with children grow up with both biological parents [19]. However, the sugar factor was excluded from the final multiple regression model. One possible explanation could be a lack of details on the dietary questions for this specific population in the questionnaire survey, which was used for the general population in the fourth National Oral Health Survey conducted in 2015 [16]. There were six scales of answers for sugar or added sugary intake-related questions (seldom, 1–3 times per month, once per week, 2–6 times per week, once per day and more than twice per day). In a rural area such as Luchuan County, the sweet consumption is relatively low compared to the national average. Therefore, a suitable specially designed questionnaire is needed for future studies.

This cross-sectional observational study is one of the few studies focusing on evaluating the effect of parental migration on dental disease outcome and oral health-related behaviors of school-aged children left behind in China. Based on the sufficient randomly selected sample size and multiple regression analyses approach, the study provides new evidence to support the potential association between parental migration and poor oral health and urgent intervention needs for the LBC population. There were several limitations in this study. Firstly, it is a cross-sectional study, and therefore cannot be used to interpret cause-effect relationships between parental migration and children’s dental caries or oral health related behaviors. Since it was a cross-sectional study, and we can’t measure how the caries experience before parental migration would impact the children’s later caries experience. Secondly, in addition to the imprecise questionnaire survey, the demographic and oral health-related behavioral questions were self-reported by the children, which could be prone to error. Thirdly, the final regression model, including gender, age, and parental migration can only explain 5.2% of caries variation, indicating that caries development and caries risk prediction indeed involves multiple genetic, biological, microbial, environmental, and socio-behavioral factors [18, 32]. Moreover, the China Women’s Federation reported that almost 20% of LBC’s parents leave home before their children’s first birthday. A significant portion of the LBC was left behind during first 3 months of their life without adequate breast feeding [3]. It is not clear how the timing of a mother’s migration affects the development of a baby’s immune system and their risk not only for oral diseases but also to their general health status. Further studies are needed to investigate those questions.

## Conclusions

In summary, our study provides new evidence that left-behind school-aged children in the countryside of Southern China are vulnerable and at an increased risk of dental caries.

## Additional files

Additional file 1: **Table S1.** Comparisons of oral health-related variables between various experiences of parental migration (*N* = 1085). (XLS 33 kb)
